# Repetitive transcranial magnetic stimulation for treatment-resistant depression in Singapore: a retrospective, naturalistic study

**DOI:** 10.3389/fpsyt.2026.1924135

**Published:** 2026-10-12

**Authors:** Yi Hang Tay, Ern Wei Christabel Thng

**Affiliations:** Department of Psychiatry, Khoo Teck Puat Hospital, Singapore, Singapore

**Keywords:** major depressive disorder, naturalistic study, neuromodulation, repetitive transcranial magnetic stimulation, theta-burst stimulation, treatment-resistant depression

## Abstract

**Introduction:**

Naturalistic repetitive transcranial magnetic stimulation (rTMS) outcome data for treatment-resistant depression remain limited in Southeast Asia. This retrospective study describes such outcomes from a general hospital in Singapore.

**Method:**

28 outpatients received rTMS (10Hz high-frequency or intermittent theta-burst) targeting the left dorsolateral prefrontal cortex (F3 scalp position) between May 2022 and May 2025. Treatment-resistant depression was defined as failing ≥2 antidepressant classes for ≥6 weeks each. The primary outcome was change in the Montgomery-Åsberg Depression Rating Scale (MADRS) score; secondary outcomes included the Quick Inventory of Depressive Symptomatology-Self Report (QIDS-SR16), the Depression, Anxiety and Stress Scale-21 (DASS-21), the Quality of Life, Enjoyment and Satisfaction Questionnaire-Short Form (Q-LES-Q-SF), the Clinical Global Impression-Severity (CGI-S), and the Global Assessment of Functioning (GAF).

**Results:**

Despite 85.7% of patients having failed ≥3 antidepressant classes, the mean MADRS score decreased by 15.8 (SD 9.1), from 29.6 (SD 7.6) to 13.9 (SD 11.1, p<0.001). This corresponded to a 67.9% response rate and a 57.1% remission rate. All secondary measures (QIDS-SR16, DASS-21, Q-LES-Q-SF, CGI-S, and GAF) showed significant improvements (all p<0.001). Shorter episode duration and higher baseline quality of life were significantly associated with remission.

**Conclusion:**

We report favourable response (67.9%) and remission (57.1%) rates within a general hospital cohort in Singapore following standardised rTMS targeting the left dorsolateral prefrontal cortex (approximated by the F3 position), despite extensive prior antidepressant medication failure. These findings contribute real-world data to the limited Southeast Asian literature on standardised rTMS protocols for treatment-resistant depression in general hospital and similar clinical settings.

## 1 Introduction

Major depressive disorder (MDD) is a leading cause of disability worldwide, with significant personal, social, and economic burden (1). In Singapore, the lifetime prevalence of depression is approximately 6.3% (2). Despite the availability of evidence-based pharmacological and psychological interventions, approximately 30 to 55% of patients with MDD fail to respond to two or more antidepressant trials of adequate dose and duration, representing a significant clinical challenge (3).

Repetitive transcranial magnetic stimulation (rTMS) is a non-invasive neuromodulation procedure involving the application of magnetic pulses to specific cortical regions of the brain, which has emerged as an important treatment option for treatment-resistant MDD. Robust evidence supports targeting the left dorsolateral prefrontal cortex (DLPFC), which can be approximated by the F3 scalp position (4, 5). Following US Food and Drug Administration (FDA) clearance in 2008, rTMS has been increasingly incorporated into clinical practice, with an established safety and efficacy profile (6). Bibliometric analyses demonstrate exponential growth in rTMS research over the past two decades, alongside increasing clinical application (7, 8). A 2023 meta-analysis of randomised, double-blinded, sham-controlled trials reported risk ratios of 2.25 and 2.78 for response and remission respectively, following rTMS in treatment-resistant MDD (9).

In Singapore, the College of Psychiatrists endorsed the use of rTMS for treatment-resistant MDD in 2014 (10), and the treatment is now available in both general hospitals and tertiary psychiatric hospitals. Khoo Teck Puat Hospital (KTPH) is a general hospital serving more than 550,000 residents in northern Singapore (11), with a Department of Psychiatry providing comprehensive outpatient and consultation-liaison mental health services. Since May 2022, the department has been providing rTMS for patients with treatment-resistant MDD using standardised, 10Hz high-frequency rTMS (HF-rTMS) and intermittent theta-burst stimulation (iTBS) protocols, both targeting the F3 scalp position as a standardised approximation of the left DLPFC.

A naturalistic study from Singapore’s sole tertiary psychiatric hospital, the Institute of Mental Health (IMH), reported response and remission rates of 20.8% and 17.0% respectively, among 58 patients with MDD treated with rTMS (12). To the best of our knowledge, this is the only study from Southeast Asia to report naturalistic rTMS outcomes. Further research in this population is important, as patient profiles in Southeast Asia differ not only from Western cohorts but also from other Asian populations, such as East Asians, in ways potentially relevant to treatment response and generalisability. These include distinct metabolic phenotypes and body composition, including more pronounced beta-cell dysfunction and higher body fat at a given body mass index (BMI) (13), which are of interest given that metabolic markers such as BMI, insulin resistance, and lipid profile have themselves been directly implicated as predictors of rTMS response (14). Depressive symptom network structure has also been shown to differ from both Western and East Asian samples (15). No published data exist on rTMS outcomes from a general hospital setting in Singapore or Southeast Asia, where clinical pathways may also differ from tertiary psychiatric hospitals, as general hospitals may be treating patients earlier in their treatment journey. Our standardised use of stimulation protocols targeting solely the left DLPFC (approximated by the F3 position) further provides an opportunity to evaluate outcomes using a consistent treatment target (4, 5).

The primary aim of this study was to describe the demographic and clinical characteristics, treatment outcomes and tolerability of rTMS among patients with MDD treated at KTPH between May 2022 and May 2025. We hypothesised that treatment outcomes in our cohort would be comparable to those reported in international naturalistic studies of rTMS for treatment-resistant depression.

## 2 Methods

### 2.1 Study design and participants

We conducted a naturalistic, retrospective cohort study of all patients who received rTMS for MDD at KTPH between May 2022 and May 2025. Patients were included if they: (1) had a primary diagnosis of MDD based on the Diagnostic and Statistical Manual of Mental Disorders, Fifth Edition (DSM-5) confirmed by their attending psychiatrist; (2) were aged ≥21 years; (3) had a baseline MADRS assessment; and (4) underwent at least one rTMS session. Exclusion criteria were: (1) inability to provide informed consent for rTMS; (2) active substance use disorder in the preceding 12 months; (3) acute suicidality; (4) personal history of epilepsy; (5) foreign metal bodies or implanted electronic devices in the head and neck; and (6) pregnancy. The criteria reflected routine clinical eligibility requirements, while baseline MADRS was additionally required for study inclusion.

The study was approved by the National Healthcare Group Domain Specific Review Board (Reference 2025-0946).

### 2.2 rTMS protocol

All treatments were delivered with the MagPro X100 stimulator and Cool D-B80 coil (Tonica Elektronik A/S [MagVenture], Farum, Denmark). Both the stimulator and coil are registered with Singapore’s Health Sciences Authority (Class B medical device; Product Number DE0019069) for the treatment of MDD since 2017, spanning our study period (16). Patients received either HF-rTMS or iTBS protocols, both targeting the F3 scalp position as a standardised approximation of the left DLPFC. The F3 position was identified by the Beam F3 method based on nasion, inion and bilateral tragus landmarks (17). The coil handle was angled 45 degrees from the midline, pointing postero-laterally, held perpendicular to the scalp, and secured with a positioning arm. Resting motor threshold (RMT) was determined at the initial session by identifying the minimum stimulation intensity required to produce visible contralateral thumb abduction for at least 50% (5/10) of consecutive stimulations over the left primary motor cortex. RMT was determined by a trained psychiatrist at the start of treatment and repeated when clinically indicated, including after medication changes, any alcohol use, or >50% deviations in sleep duration or caffeine intake from baseline. All patients reported abstinence from alcohol throughout treatment.

The HF-rTMS protocol consisted of 10Hz stimulation in 4-second trains repeated every 15 seconds, delivering 3000 pulses per session at 120% RMT over 18.5 minutes. The iTBS protocol consisted of triplet 50Hz bursts repeated at 5Hz (theta frequency) in 2-second trains repeated every 10 seconds, delivering 600 pulses at 120% RMT over 3 minutes and 9 seconds. Both protocols received US FDA clearance for MDD in 2017 and 2018 respectively using standard figure-of-eight coils; clearance specifically for the Cool D-B80 coil (used in our service) followed in 2025 (18–20). There is no protocol-specific regulation for rTMS in Singapore, although our local College of Psychiatrists released a position paper in 2014 supporting the use of protocols at frequencies 1Hz and above, targeting the left or right DLPFC (10).

The standard acute course consisted of weekday sessions for 3 to 6 weeks, initially comprising 20 to 30 sessions. Treatment could be extended after session 20 in 10-session increments if MADRS-defined remission had not been achieved, typically resulting in 30–40 sessions. Patients receiving HF-rTMS with <50% improvement in MADRS after 20 or 30 sessions were offered the option of switching to iTBS, as a pragmatic clinical approach to non-responders in a standard-length course of HF-rTMS. This switching strategy was guided by consensus recommendations to consider prior non-response to one rTMS protocol when selecting an alternative protocol for a given patient (5). For a handful of patients, treatment intensity was gradually increased from 80% to 120% of RMT over the first 2 to 4 sessions for tolerability; all patients subsequently received 120% RMT. Trained nursing staff monitored all sessions, with psychiatrist support available for adverse events. No tapering schedule was used.

### 2.3 Outcome measures

The primary outcome was change in clinician-rated MADRS (21) from baseline to the end of the acute treatment course. MADRS is a 10-item clinician-administered measure of depressive symptom severity ranging from 0 to 60. Response was defined as ≥50% reduction from baseline, and remission was defined as a final MADRS score ≤10.

Secondary outcomes included: (1) the Quick Inventory of Depressive Symptomatology-Self Report, 16-item version (QIDS-SR16) (22), a self-report measure of depression severity, with scores ranging from 0 to 27; (2) Depression, Anxiety and Stress Scale-21 (DASS-21) (23), a 21-item self-report questionnaire assessing depression, anxiety, and stress symptoms; (3) Quality of Life, Enjoyment and Satisfaction Questionnaire-Short Form (Q-LES-Q-SF) (24), a 16-item self-report measure of quality of life and life satisfaction; (4) Clinical Global Impression-Severity (CGI-S) (25), a single-item clinician-rated scale of overall illness severity ranging from 1 (normal) to 7 (among the most extremely ill); and (5) Global Assessment of Functioning (GAF) (26), a clinician-rated measure of overall psychological, social, and occupational functioning on a scale from 0 to 100. DASS-21 subscale scores were multiplied by two as per standard scoring instructions (23). Q-LES-Q-SF scores were calculated as (total raw score of the 14 core items − 14)/56, expressed as a percentage (24). Although GAF was removed from DSM-5 partly because of low inter-rater reliability (27), we retained it for its clinical utility and ease of interpretation, for assessing functional change alongside symptomatic improvement. All outcome measures were administered at baseline (within one week before commencing rTMS) and at the completion or termination of the acute treatment course (end-point). MADRS, QIDS-SR16, and DASS-21 were additionally assessed at session 12.

### 2.4 Clinical data collection

Demographic and clinical data extracted from electronic medical records included age, gender, ethnicity, educational level, employment status, family history of mental illness, duration of current depressive episode, number of previous depressive episodes, number of failed antidepressant medications and classes, current psychotropic medications, psychiatric hospitalisation, and prior ECT. Treatment-resistant MDD was defined as failure to respond to at least two antidepressant medication trials from different pharmacological classes at adequate dose and duration of at least 6 weeks each. Medication adherence was assessed based on clinician documentation and assumed in the absence of evidence to the contrary. rTMS treatment data included protocol type, number of sessions, protocol switching, side effects and adverse events.

### 2.5 Statistical analysis

Continuous variables were summarised as means and standard deviations (SD) or medians and interquartile ranges (IQR) based on distribution properties, and categorical variables as frequencies (%). Normality of within-participant change scores was assessed using the Shapiro-Wilk test. Paired t-tests with Cohen’s d were used for normally distributed outcomes; the Wilcoxon signed-rank test with matched-pairs rank-biserial correlation (r) was used for CGI-S. Exploratory univariate analyses examined demographic and clinical associations with treatment outcomes and were intended to be hypothesis-generating. Given the small sample size, the study was insufficiently powered for multivariate regression. Spearman correlation was used for continuous and ordinal variables because several variables, including antidepressant counts and episode duration, were not normally distributed; it provided a simple, non-parametric measure of the direction and strength of monotonic associations. Fisher’s exact test was used for gender given small expected cell counts. Analyses were uncorrected for multiple comparisons. Statistical analyses were performed using IBM SPSS Statistics v32.0 (IBM Corp, Armonk, NY, USA), with two-tailed p<0.05 considered statistically significant.

For all patients who discontinued treatment early, end-point scales were administered at treatment termination. All but two patients had complete baseline and endpoint assessments across all outcome measures, as well as complete session-12 assessments for MADRS, QIDS-SR16, and DASS-21. The two patients who did not have interim and endpoint assessments had terminated their treatment after four sessions, due to possible hypomania and time commitment reasons respectively. Both were included in primary and secondary outcome analyses using last observation carried forward (LOCF), with baseline scores carried forward as interim and endpoint values, and both patients classified as non-responders. There was no other missing data. All patients were included in the analyses of primary and secondary outcomes.

## 3 Results

### 3.1 Patient demographics and clinical characteristics

A total of 28 patients with MDD received rTMS treatment at KTPH between May 2022 and May 2025 and met the eligibility criteria. **Table 1** displays the patient demographics and characteristics. The cohort had a mean age of 43.4 years (SD 16.4), was 57.1% female, and reflected Singapore’s ethnic population distribution. All patients met criteria for treatment resistance, and 85.7% (n=24) failed at least three antidepressant classes. Baseline DASS-21 subscale scores reflected substantial depression (30.8, SD 9.4), anxiety (20.3, SD 10.3), and stress (25.6, SD 9.5) symptom burden. The remaining baseline assessment scores are reported in **Table 2**.

**Table 1:** Patient demographics and clinical characteristics (n=28).

Clinical characteristic	n(%)
Age in years, mean (SD)	43.4 (16.4)
Gender	
Male, n (%)	12 (42.9)
Female, n (%)	16 (57.1)
Race	
Chinese, n (%)	24 (85.7)
Malay, n (%)	2 (7.1)
Indian, n (%)	1 (3.6)
Eurasian, n (%)	1 (3.6)
Employment status	
Full-time, n (%)	8 (28.6)
Part-time, n (%)	2 (7.1)
National service, n (%)	1 (3.6)
Retired, n (%)	4 (14.3)
Unemployed, n (%)	13 (46.4)
Highest educational level in years, median (IQR)	17.0 (13.0-17.0)
Marital status	
Single, n (%)	14 (50.0)
Married, n (%)	12 (42.9)
Divorced, n (%)	2 (7.1)
Smoking status	
Non-smoker, n (%)	20 (71.4)
Current smoker, n (%)	8 (28.6)
Alcohol history	
None, n (%)	18 (64.3)
Less than once a week, n (%)	10 (35.7)
Drug history	
None, n (%)	26 (92.9)
Past drug use, n (%)	2 (7.1)
Living arrangement	
Lives with family, n (%)	21 (75.0)
Lives alone, n (%)	7 (25.0)
Family history of mental illness	
No, n (%)	17 (60.7)
Yes, n (%)	11 (39.3)
MDD subtype	
None, n (%)	15 (53.6)
With anxious distress, n (%)	8 (28.6)
With melancholic features, n (%)	4 (14.3)
With psychotic features, n (%)	1 (3.6)
Secondary diagnosis	
None, n (%)	14 (50.0)
Dysthymia, n (%)	9 (32.1)
Posttraumatic stress disorder, n (%)	2 (7.1)
Borderline personality disorder, n (%)	1 (3.6)
Obsessive-compulsive disorder, n (%)	1 (3.6)
Social anxiety disorder, n (%)	1 (3.6)
Age of onset of depression in years, median (IQR)	24.0 (18.3-40.0)
Number of major depressive episodes, median (IQR)	3.0 (3.0-3.8)
Duration of current depressive episode in months, median (IQR)	12.0 (4.0-24.0)
History of ECT	
No, n (%)	27 (96.4)
Yes, n (%)	1 (3.6)
History of TMS	
No, n (%)	23 (82.1)
Yes, n (%)	5 (17.9)
Number of antidepressant trials, median (IQR)	5.0 (3.3-6.8)
Number of antidepressant classes, median (IQR)	3.0 (3.0-4.0)
Number of failed antidepressants, n (%)	
4 or more	21 (75.0)
3	6 (21.4)
2	1 (3.6)
Number of failed antidepressant classes, n (%)	
4 or more	11 (39.3)
3	13 (46.4)
2	4 (14.3)
Current psychotropic medications	
Antidepressant monotherapy, n (%)	3 (10.7)
Combination of antidepressants excluding mood stabiliser(s) and antipsychotic(s), n (%)	18 (64.3)
Combination with antipsychotic(s), n (%)	10 (35.7)
Combination with lithium, n (%)	2 (7.1)
Combination with mood stabiliser(s) excluding lithium, n (%)	3 (10.7)
Combination with regular benzodiazepine and/or Z-drug, n (%)	9 (32.1)
Number of Treatment Sessions, median (IQR)	30.0 (23.3-30.0)
Final rTMS protocol at the last treatment session	
HF-rTMS, n (%)	26 (92.9)
iTBS, n (%)	2 (7.1)

**Table 2:** Primary and secondary outcomes.

Assessment scale	N	Pre-rTMS, mean (SD) or median (IQR)	Session 12, mean (SD)	End of acute rTMS course, mean (SD) or median (IQR)	Mean paired difference (95% CI)^a^	Test statistic^a^	P-value^a^	Effect size^a^
MADRS	28	29.6 (7.6)	20.4 (10.7)	13.9 (11.1)	-15.8 (-19.3, -12.2)	t=-9.205	<0.001**	d=-1.74
QIDS-SR16	28	16.5 (5.6)	12.2 (6.5)	10.5 (7.2)	-5.9 (-8.4, -3.5)	t=-5.002	<0.001**	d=-0.95
DASS-21	28	76.6 (25.2)	51.9 (34.0)	47.4 (35.7)	-29.2 (-38.6, -19.8)	t=-6.372	<0.001**	d=-1.20
Q-LES-Q-SF	28	33.8 (14.3)	Not assessed^b^	47.3 (22.2)	13.5 (8.1, 19.0)	t=5.071	<0.001**	d=0.96
CGI-S	28	4.0 (4.0-5.0)	Not assessed^b^	2.5 (2.0-5.0)	Not applicable (Wilcoxon)	Z=-4.33	<0.001**	r=0.82
GAF	28	51.8 (4.6)	Not assessed^b^	70.9 (15.6)	19.1 (13.7, 24.5)	t=7.246	<0.001**	d=1.37

**p ≤ 0.01.

^a^ Pre-rTMS vs. End of acute rTMS course; session-12 values are descriptive only and not included in this comparison.

BQ-LES-Q-SF, CGI-S, and GAF were assessed only at baseline and end of acute treatment; no session-12 measure was collected for these scales.

### 3.2 Treatment parameters

All patients received standardised rTMS targeting the left DLPFC, approximated by the F3 position. Most (92.9%, n=26) received HF-rTMS, 1 received iTBS, and 1 received HF-rTMS initially, before switching to iTBS after 30 sessions. The median number of treatment sessions was 30.0 (IQR 23.3-30.0). **Figure 1** shows the flowchart of the patients screened and evaluated.

**Figure 1:**
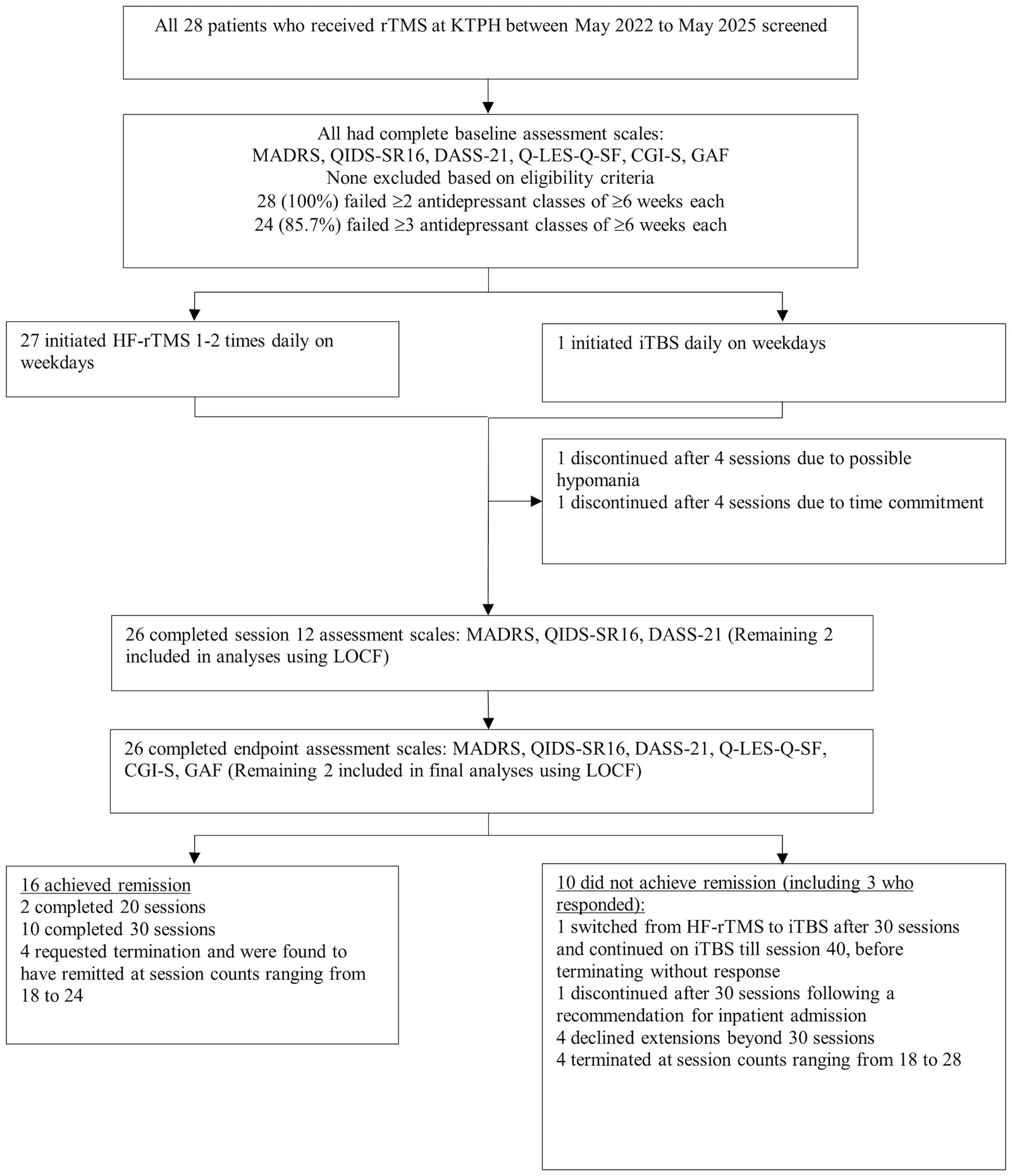
Flowchart of patients screened.

### 3.3 Primary outcomes

MADRS scores decreased significantly from 29.6 (SD 7.6) to 13.9 (SD 11.1), representing a mean change of −15.8 points (SD 9.1, 95% CI −19.3 to −12.2, p<0.001; Cohen’s d=−1.74), or a mean reduction of 55.8% from baseline (calculated as the mean of individual percentage reductions). The MADRS response rate was 67.9% (n=19, 95% CI: 49.3-82.1%), and remission rate was 57.1% (n=16, 95% CI: 39.1-73.5%).

### 3.4 Secondary outcomes

All secondary outcome measures showed statistically significant improvements. Details of primary and secondary outcomes are displayed in **Table 2**.

### 3.5 Exploratory analyses: factors associated with response and remission

Exploratory univariate analyses examined associations between selected baseline demographic and clinical variables, and treatment response and remission (**Table 3**). Response was significantly associated with lower baseline depression severity across clinician-rated and self-reported measures, and higher baseline quality of life. Remission was similarly associated with lower baseline depression severity, higher baseline quality of life, and shorter current major depressive episode duration. Age, gender, number of failed antidepressant trials, and number of rTMS sessions were not significantly associated with either outcome. As these analyses were exploratory and uncorrected for multiple comparisons, findings should be considered hypothesis-generating.

**Table 3:** Exploratory **c**orrelates of **r**esponse and **r**emission on MADRS **f**ollowing rTMS^a^.

Variable	Response on MADRS		Remission on MADRS	
	Spearman r/Fisher’s exact test	P-value	Spearman r/Fisher’s exact test	P-value
Age	0.019	0.924	-0.072	0.717
Gender	-^b^	1.000	-^b^	0.702
Duration of current major depressive episode	-0.295	0.127	-0.445	0.018*
Number of failed antidepressant trials	0.346	0.072	0.218	0.266
Number of rTMS sessions	0.258	0.185	0.083	0.676
Baseline MADRS	-0.399	0.036*	-0.573	<0.001**
Baseline QIDS-SR16	-0.461	0.014*	-0.488	0.008**
Baseline DASS-21	-0.427	0.024*	-0.532	0.004**
Baseline Q-LES-Q-SF	0.626	<0.001**	0.707	<0.001**

*p ≤ 0.05; **p ≤ 0.01.

^a^ All analyses are exploratory, uncorrected for multiple comparisons, and for hypothesis-generating only. The risk of false-positive findings is high and results should not be interpreted as confirmatory.

^b^ Fisher’s exact test as opposed to χ² due to small expected cell counts (n<5 in one or more cells).

### 3.6 Safety and tolerability

rTMS was generally well-tolerated. While adverse effects were actively recorded throughout the treatment course, they predominated in the first week. The most common were mild headache (32.1%, n=9), scalp discomfort or pain (28.6%, n=8), and facial muscle twitching (14.3%, n=4); these were transient, mild, and resolved spontaneously or with simple analgesia without necessitating treatment discontinuation. No tinnitus, hearing impairment, syncope, or seizure was reported.

Two events led to treatment discontinuation on the assessing psychiatrist’s recommendation. One patient developed possible hypomanic symptoms - elevated mood and a subjective experience of racing thoughts - after four sessions of HF-rTMS, against a background of poor sleep in the preceding three weeks. The treating psychiatrist could not determine whether the symptoms reflected treatment-emergent hypomania, an early positive response to rTMS, or recent life events. Following discussion with the patient, a shared decision was made to terminate treatment as a precautionary measure. The diagnosis was not revised to bipolar disorder, and symptoms resolved within two weeks without any medication adjustment. A second patient with pre-existing passive suicidal ideation developed active suicidal ideation after 30 sessions; rTMS was discontinued and inpatient admission recommended for risk management and medication optimisation. The treating psychiatrist considered an acute psychosocial stressor more likely responsible; however, a causal contribution from rTMS cannot be definitively excluded in either case.

## 4 Discussion

Our naturalistic study is the first to report response (67.9%) and remission (57.1%) rates following rTMS for treatment-resistant MDD in a general hospital setting in Singapore. These rates are consistent with large naturalistic studies of rTMS delivered in routine clinical practice (28, 29), including a multi-site United States registry of over 5,000 patients with MDD reporting response rates of 58-83% and remission rates of 28-62% (29). A recent meta-analysis of randomised controlled trials reported pooled response and remission rates of nearly 40% and 36% respectively, among patients who received rTMS after failing at least two adequate antidepressant trials (9). Taken together, our findings are in keeping with real-world therapeutic outcomes reported for rTMS in MDD internationally.

In the landmark STAR*D study, MDD remission rates declined substantially with each successive pharmacological treatment, falling to 13.7% and 13.0% following the third and fourth antidepressant medication trials respectively (30). This diminishing return with sequential pharmacotherapy underscores the challenge of finding suitable treatments in the face of treatment resistance. Direct comparison of treatment outcome rates cannot be made due to differences in study design and population. However, it is against this backdrop that favourable treatment outcomes achieved by rTMS in cohorts such as ours strengthen support for guidelines recommending rTMS as a first-line treatment for patients with treatment-resistant MDD (31).

Improvements across all secondary outcome measures reinforce the clinical meaningfulness of the treatment response. This included DASS-21 subscales; however, given the absence of a control group and overlap among depressive, anxiety and stress symptoms, these findings should be interpreted as concomitant changes in symptom domains that frequently co-occur with depression, rather than as evidence of an independent effect on anxiety or stress. While evidence for rTMS in generalised anxiety disorder is mixed (32), the relief of anxiety symptoms in anxious depression is being increasingly recognised (33).

Shorter duration of current major depressive episode was associated with better remission outcomes, consistent with previous findings (34, 35), including in Asian populations (36), although it was not significantly associated with response. This raises the possibility that while patients with more chronic depression may experience greater difficulty achieving remission, they could still benefit from substantial symptom reduction. Higher quality of life at baseline was significantly associated with both response and remission in our study, extending previous work on quality of life and rTMS outcomes (37). These associations were uncorrected for multiple comparisons and remain hypothesis-generating; confirmation in larger, prospectively-designed studies is needed.

The findings below warrant similar interpretive restraint. Number of rTMS sessions was not associated with remission, in line with previous studies (38). Lower baseline depression severity was strongly associated with remission, consistent with prior reports (34, 39), but this requires particular caution because baseline MADRS is mathematically coupled to the remission definition (MADRS ≤ 10 at endpoint). A lower baseline MADRS mechanically reduces the change required to cross this threshold, independent of treatment effect. The association should therefore be interpreted as an artifact of this coupling rather than evidence of a clinically meaningful relationship. Baseline MADRS, QIDS-SR16, and DASS-21 are also inherently collinear, capturing overlapping depressive and general-distress symptom burden, so their individually significant associations with the outcomes should not be considered as independent lines of evidence.

Response and remission rates vary considerably across naturalistic studies because of differences in patient populations and treatment delivery. As previously noted, the only other published naturalistic rTMS outcomes from Southeast Asia come from Singapore’s tertiary psychiatric hospital, IMH, which reported a response rate of 20.8% and remission rate of 17.0% (12). Baseline MADRS was comparable to ours (28.1 [SD 7.3] at IMH vs. 29.6 [SD 7.6] at KTPH), but our study designs and multiple cohort and treatment differences limit comparison of the outcomes. More patients in our cohort had failed three or more antidepressant medications (96.4%, vs 66.2% in the IMH study). Our cohort was treated as outpatients, whereas 40.9% of the IMH cohort were inpatients, a difference in care settings that further constrains comparison. Only one patient in our cohort had prior ECT exposure, compared with 21.2% at IMH; evidence on how prior ECT relates to subsequent rTMS outcomes is mixed (40–43), and this difference in ECT history represents a further point of divergence between the cohorts. Notably, only 55.2% of the IMH cohort received rTMS at the left DLPFC approximated by the F3 position - the stimulation site with the strongest evidence for efficacy in treating depression (5, 44, 45) - compared to 100% of our cohort. Given these differences, the small sample sizes, and the retrospective, naturalistic designs of both studies, any interpretations regarding the numerically different treatment outcomes cannot be reliably drawn.

### 4.1 Strengths and limitations

Our strengths include the follow-up with both clinician-administered and self-reported depression rating scales to verify trend alignment before drawing conclusions, our exclusive use of standardised protocols targeting the evidence-based left DLPFC as approximated by the F3 position (minimising protocol heterogeneity), and a cohort consisting exclusively of patients meeting our treatment-resistant MDD definition of failure to respond to ≥2 antidepressant medications from different pharmacological classes at adequate dose and duration (≥6 weeks each) - a subgroup less frequently examined in naturalistic rTMS studies (28, 46–51) despite its direct relevance to clinical guidelines (3).

Several limitations should be acknowledged. First, owing to the retrospective design, all patients continued baseline psychotropic medications throughout rTMS treatment, without controlling for potential medication effects. Protocol selection was based on clinician’s recommendation, personal preference, and tolerability. However, this approach reflects real-world clinical practice and enhances generalisability. Second, the interim assessment at session 12 was a fixed rather than proportional assessment timepoint because treatment duration varied between patients, limiting its interpretation. Third, the small sample size precluded multivariate regression analyses. Presented associations should not be interpreted as predictive biomarkers without confirmation in larger prospective studies. Fourth, the Beam F3 method provides a standardised approximation of the left DLPFC rather than an individualised coordinate achievable through neuronavigation. Fifth, LOCF was used for two patients who discontinued after four sessions. This may have biased estimates of treatment effect, although its impact on the overall findings is likely limited given that this applied to only two patients. Sixth, longer-term outcomes and maintenance strategies were not examined; future studies should clarify the durability of response. Finally, the absence of a control group precluded definitive attribution of outcomes to rTMS versus the natural course of illness.

## 5 Conclusion

This naturalistic study found favourable response and remission rates in a Singapore general hospital cohort of patients with treatment-resistant MDD receiving standardised F3-targeted rTMS, in line with international benchmarks and contributing to the limited rTMS outcome literature from Southeast Asia. Exploratory analyses found that higher baseline quality of life and shorter duration of the current major depressive episode were associated with higher remission rates, although these findings remain hypothesis-generating and require confirmation in larger studies. Future research employing prospective designs with longer follow-up could clarify optimal maintenance strategies.

## Data Availability

The datasets presented in this article are not readily available because of ethical restrictions and institutional governance policies regarding participant confidentiality and risk of re-identification. Requests to access the datasets should be directed to the NHG Health Domain Specific Review Board at nhggroup.OHRPP@nhghealth.com.sg. Further inquiries can be directed to the corresponding author.
